# Oxidative Stress and Rare Diseases: From Molecular Crossroads to Therapeutic Avenues

**DOI:** 10.3390/antiox10040617

**Published:** 2021-04-16

**Authors:** Carlos Romá-Mateo, José Luis García-Giménez

**Affiliations:** 1 Department of Physiology, Faculty of Medicine and Dentistry, University of Valencia, 46010 Valencia, Spain; 2 INCLIVA Biomedical Research Institute, 46010 Valencia, Spain; 3 Center for Biomedical Network Research on Rare Diseases (CIBERER), Institute of Health Carlos III, Valencia, Spain; 4 EpiDisease SL (Spin-Off Ciber-ISCIII), Paterna, 46980 Valencia, Spain

Writing an editorial about rare diseases can become a messy subject from the biological perspective. Unlike other nosological entities, the term “rare diseases” does not stem from a series of biological features, but from a unique common trait which, besides, is a rather arbitrary criterion: the requisite for a disease to be rare is epidemiological, and classification varies throughout different countries. The only objective fact that could be assumed as the cause for the low prevalence found for these pathologies could be their monogenic and mendelian pattern of heritability; and even this is not always the case in up to 20% of the cases of rare diseases which have a multifactorial, sometimes idiopathic origin. Thus, we are left with only the epidemiological criteria to decide whether a particular disease falls within or outside of the definition. From the perspective of biomedical research, we face a complex, heterogeneous group of entities which can only be classified based on very broad and unspecific categories. Moreover, rare diseases are commonly associated with phenotypic variability throughout the natural history of disease, due to diverse molecular events which contribute over the years to the worsening of any condition. So, these particular features of rare diseases raise the critical following question: at the molecular level, what do they have in common? Is there a common biological link suitable to become a clinical target? Could we design therapeutic approaches to address the symptoms of more than one type of disease?

As very often happens in science, for a complex question like this… there is not a simple answer. However, the study of oxidative stress at the cellular level might hold some of the key clues, as proves the collection of papers presented in our Special Issue “Oxidative Stress and Rare Diseases”. When gazing at the compilation of works included, one can find a somehow paradoxical relationship between them all: even though oxidative stress still harbors a central role in the understanding of the pathophysiology of rare diseases, the way researchers are approaching it seems to be shifting. Evidence of the presence of free radicals (i.e., reactive oxygen species or nitrogen reactive species) and their products (i.e., oxidized nucleic acids, proteins and lipids), or the imbalance in antioxidant systems, is abundant and beyond doubt. Nonetheless, it was always difficult to assess causative links between free radicals and their subproducts and the symptoms of disease; and in a similar way, antioxidant-based therapies have failed to provide the initially expected cure for those conditions in which these signs were so obvious. The never-ending chicken-egg story, so to speak. This fact should not discourage researchers: oxidative stress keeps playing a key role in the maintenance of cellular homeostasis, and even more, our understanding of cellular signaling pathways and their adaptation capacity to environmental changes has been deeply enriched by the analysis of oxidative stress and antioxidant mechanisms.

Lessons learned over the last decades are mirrored by the present collection of works, which includes a diverse group of pathologies ranging from Fanconi anemia [1], neuromuscular diseases like Friedreich’s ataxia [2,3] and Huntington’s disease [4], to retinal dystrophies [5]. The neuronal degeneration associated with these latter examples is reviewed by Espinós and collaborators [6], in a thorough revision which references the impact of oxidative stress research in a large list of neurodegenerative disorders that include rare ataxias, diseases with iron accumulation, the aforementioned rare neuromuscular disorders, retinal dystrophies and different rare epilepsies. Assuming oxidative stress as a common trait, we can also conclude that mitochondrial impairment (together with alterations in mitophagy), deficiencies in iron metabolism, decreased glutathione levels and importantly, an increase in neuroinflammation, are all contributing factors that worsen and accelerate disease progression in the vast majority, if not all, of those rare diseases reviewed. This fine dissection of the role of free radicals and antioxidant systems in the interplay with inflammatory pathways is sustained by ongoing therapies or pilot studies [4,5]: therapeutic strategies are widening the use of antioxidant drugs [2,3,5], and starting to combine them with pharmacological manipulation of inflammation and proteostasis, as well as mitoprotective strategies [6].

Are we then facing a shift in the paradigm of oxidative stress research? In our opinion, there is no need to make such dramatic statements. This is just a natural progression in scientific knowledge: accumulating evidence leads to the finding of interconnections and commonalities that help to figure out the bigger picture. From a molecular biology perspective, the road built upon free radicals and antioxidant pathways and regulators has led to a crossroads in which inflammation, proteostasis, iron metabolism and free radicals are no longer to be studied as independent players, but as part of a series of feedback mechanisms that we are, at the present time, more able to visualize and understand. We should be aware that crossroads can be tricky and confusing, but as long as we do not miss directions, the translation of this new landscape into a series of more effective therapies that improve the development of precision medicine might just be a matter of time.
